# Integrity Concept for Maritime Autonomous Surface Ships’ Position Sensors †

**DOI:** 10.3390/s20072075

**Published:** 2020-04-07

**Authors:** Paweł Zalewski

**Affiliations:** Maritime University of Szczecin, Wały Chrobrego 1-2 Str., 70-500 Szczecin, Poland; p.zalewski@am.szczecin.pl

**Keywords:** MASS, maritime position sensors, integrity, PVT, PNT, GNSS

## Abstract

The primary means for electronic position fixing currently in use in majority of contemporary merchant ships are shipborne GPS (Global Positioning System) receivers or DGPS (Differential GPS) and IALA (International Association of Lighthouse Authorities) radio beacon receivers. More advanced GNSS (Global Navigation Satellite System) receivers able to process signals from GPS, Russian GLONASS, Chinese Beidou, European Galileo, Indian IRNSS, Japan QZSS, and satellite-based augmentation systems (SBAS), are still relatively rare in maritime domain. However, it is expected that such combined or multi-system receivers will soon become more common in maritime transport and integrated with gyro, inertial, radar, laser, and optical sensors, and they will become indispensable onboard maritime autonomous surface ships (MASS). To be prepared for a malfunction of any position sensors, their state-of-the-art integrity monitoring should be developed and standardized, taking into account the specificity of MASS and e-navigation safety. The issues of existing requirements, performance standards, and future concepts of integrity monitoring for maritime position sensors are discussed and presented in this paper.

## 1. Introduction

It is expected that multi-system GNSS receivers together with GNSS-independent position systems and sensors will soon become common in maritime transport and indispensable onboard maritime autonomous surface ships (MASS) as they have already been onboard many vessels engaged in offshore work. To be prepared for a malfunction of any shipborne position sensors, their state-of-the-art integrity monitoring should be developed and standardized [1], taking into account the specificity of MASS navigation safety. The International Maritime Organization (IMO) took steps to address autonomous ships in 2018 and 2019 by adopting the definition of MASS, their four degrees of autonomy, and a framework for the regulatory scoping exercise [2].

IMO has defined a MASS as a ship which, to a varying degree, can operate independent of human interaction, and organized its degrees of autonomy as follows [2]:

(1) Ship with automated processes and decision support: Seafarers are on board to operate and control shipboard systems and functions. Some operations may be automated and at times be unsupervised, but with seafarers on board ready to take control.
(2) Remotely controlled ship with seafarers on board: The ship is controlled and operated from another location. Seafarers are available on board to take control and to operate the shipboard systems and functions.
(3) Remotely controlled ship without seafarers on board: The ship is controlled and operated from another location. There are no seafarers on board.
(4) Fully autonomous ship: The operating system of the ship is able to make decisions and determine actions by itself.

To achieve safety of a MASS operation, redundant, multi-system receivers integrated with magnetometer, gyro, inertial, radar, laser and optical sensors will be necessary onboard these vessels on all four autonomy levels. However, there are still no IMO adopted integrity measures for contemporary maritime electronic position fixing systems (EPFS) such as GNSS, heading, radar or optical sensors. The current IMO performance specifications of Global Navigation Satellite System (GNSS) shipborne receivers are system-level specifications, which do not take into account threats to GNSS position-fixing that are local to the user (such as a reduced number of satellites in view due to signal obscuration, multipath, or radio interference). Furthermore, the GNSS user’s receiver is not required to implement its own integrity algorithms. However, recommendations set out in IMO resolution A.915(22) [3] together with established techniques of receiver autonomous integrity monitoring or fault-detection (RAIM, FD, FDE) can lead to construction of maritime continuity and integrity algorithms similar to the ones standardized for satellite based augmentation systems (SBAS) used in the aviation sector [4].

The aim of the work described in the paper is to develop a concept of MASS position sensors’ integrity evaluation and its presentation for the human operator/navigator. The concept is based on monitoring of integrity risk of position and heading (attitude) data that takes into account not only EPFS point positioning but also propagation of position error to ship’s body furthest points. Additionally, the idea of the presented concept is to conceptually split the integrity monitoring between two functions that, combined, could provide a modular and robust way of MASS positioning safety evaluation in line with the positioning, navigation and timing (PNT) data integrity concepts handled by IMO and other PNT working groups [3,5]:

- Integrity positioning function whose goal is to obtain a positional error bound that guarantees, with a certain probability, that the ship is within specific boundaries. It measures the integrity of the positioning at user level.
- Integrity operational navigation function which refers to how the position error bound (the protection level) is used from an operational perspective.

## 2. Related Work

There has been significant progress within the last decades in the development of Autonomous Surface Vehicles (ASV). The ASVs have been produced in a range from small „pocket” platforms that can be carried in the trunk of the car to larger units of a nearly seagoing range [6]. These vessels have been mostly used for hydrographic measurements in shallow waters or an area/environment monitoring close to shores or offshore structures. Sea or oceangoing vessels of sizes comparable to contemporary transport ships are usually not included in this group. The latter have been generally referred as MASS, following the research presented in [7,8,9,10,11,12].

The summary and conclusions from the findings of five EU projects considering the issue of MASS were presented in [8]. The big challenge envisioned for a future autonomous technology is to show that an unmanned system is at least as safe as a manned ship system, and to provide the shore control operators with adequate situation awareness. The taxonomy of MASS in terms of the operational scenario possibilities, in how the control problem is solved and how responsibilities are divided between humans and computer systems, was developed in [9]. It suggested building a standard based on autonomous cars; however, the main problem is that ships are larger and slower, but consequences of accidents may be more severe. The example research that focused on adaptive navigation of MASS in an uncertain environment was described in [7,13]. To achieve intelligent obstacle avoidance of MASS in coastal waters or a port, an autonomous navigation decision-making model based on dynamic programming method or hierarchical deep reinforcement machine learning was proposed. Correspondingly, the use of machine learning and deep learning artificial intelligence (AI) techniques as a means to integrate multiple sensor modalities into a cohesive approach to navigation for autonomous ships was presented in [13]. Finally, a System-Theoretic Process Analysis (STPA) was applied in the research presented in [11] in order to develop and analyse a preliminary model of the unmanned shipping system and elaborate safety recommendations for future developers of the actual system. The conclusion was that STPA-like software is likely to have much greater influence on future autonomous ships’ safety performance than it has on contemporary merchant vessels. 

All this research stress that at some point the availability of GNSS or generally PNT data is crucial for safe MASS operations. In the case of small ASV, the risk evolving from faulty PNT data may be of relatively low significance, but in the case of MASS the consequences can be serious.

The solution to PNT data reliability in maritime domain can be derived from other transport domains. For example, reliable aircraft guidance is one of the main contributors to the high level of safety that is achieved today on modern aircraft. Especially during the landing phase, where aircraft are close to other surrounding traffic and ground obstacles, any undetected deviation from the desired flight path may lead to catastrophic consequences. An aviation low cost system that consisted of a classical GNSS/INS system augmented by an optical positioning system for integrity monitoring was described in [10]. Key differences between integrity monitoring scheme in aviation domain and urban transport field were addressed in [14,15]. The accuracy and integrity requirements, and the positioning system for no-fly zone unmanned aerial vehicle (UAV) management were specified in [16]. All these works, together with ICAO and IMO standards or guidelines, were a basis for the development of integrity concept for MASS’ position sensors as presented further. 

## 3. Evolution of IMO GNSS Integrity Concepts

IMO started work on the integrity of GNSS as part of worldwide radio navigation systems (WWRS) in the end of 1990s together with the International Civil Aviation Organization (ICAO). This was the time of satellite-based augmentation systems (SBAS) development whose signals could be used worldwide as external source of GNSS corrections and integrity data.

Resolution A.915(22) [3] on revised maritime policy and requirements for a future GNSS was adopted by IMO in 2001. This resolution proposed, for the first time, internal (user-level or shipborne) and external (provided by external stations) integrity monitoring to maritime domain. Integrity monitoring was defined as the process of determining whether the system performance (or individual observations conducted by the system) allows its use for navigation purposes. Overall GNSS system integrity was described by three parameters: the threshold value or alert limit (*AL*), the time to alarm (*TTA*), and the integrity risk (*IR*). Definitions of the following terms were introduced to the maritime users:

- Integrity: The ability to provide users with warnings within a specified time when the system should not be used for navigation.
- Craft autonomous integrity monitoring (CAIM): this is a technique whereby various navigation sensor information available on the craft is autonomously processed to monitor the integrity of the navigation signals.
- Receiver autonomous integrity monitoring (RAIM): A technique whereby the redundant information available at a GNSS receiver is autonomously processed to monitor the integrity of the navigation signals.
- Continuity: The probability that, assuming a fault-free receiver, a user will be able to determine position with specified accuracy and is able to monitor the integrity of the determined position over the (short) time interval applicable for a particular operation within a limited part of the coverage area.
- *IR*: The probability that a user will experience a position error larger than *AL* without an alarm being raised within the specified *TTA* at any instant of time at any location in the coverage area.
- Coverage: The coverage provided by a radionavigation system is that surface area or space volume in which the signals are adequate to permit the user to determine position to a specified level of performance.
- Latency: The time lag between the navigation observations and the presented navigation solution.
- Chart error (*CE*): Position errors in the chart caused by inaccuracies in surveying and by errors in the reference geodetic system.
- Navigation system error (*NSE*): The combined error of the GNSS position estimate (*PE*) usually referenced to a common consistent reference point (CCRP) and *CE*:
  *NSE* = *CE* + *PE*. (1)
- Vessel Technical Error (*VTE*): This is the difference between the indicated craft position and the indicated command or desired position. It is a measure of the accuracy the craft is controlled with. Components are cross track error (*XTE*) and along track error (*ATE*).
- Total System Error (*TSE*): The overall navigation performance can be described by the TSE. Assuming the contributions to *TSE* from *NSE* and *VTE* are random, the *TSE* can be described as:
  *TSE*^2^ = *NSE*^2^ + *VTE*^2^. (2)
- Reliability of a position fix: A measure of the propagation of a non-detected gross error (outlier) in an observation to the position fix. This “external” reliability is usually expressed in terms of marginally detectable error.

**Figure 1:**
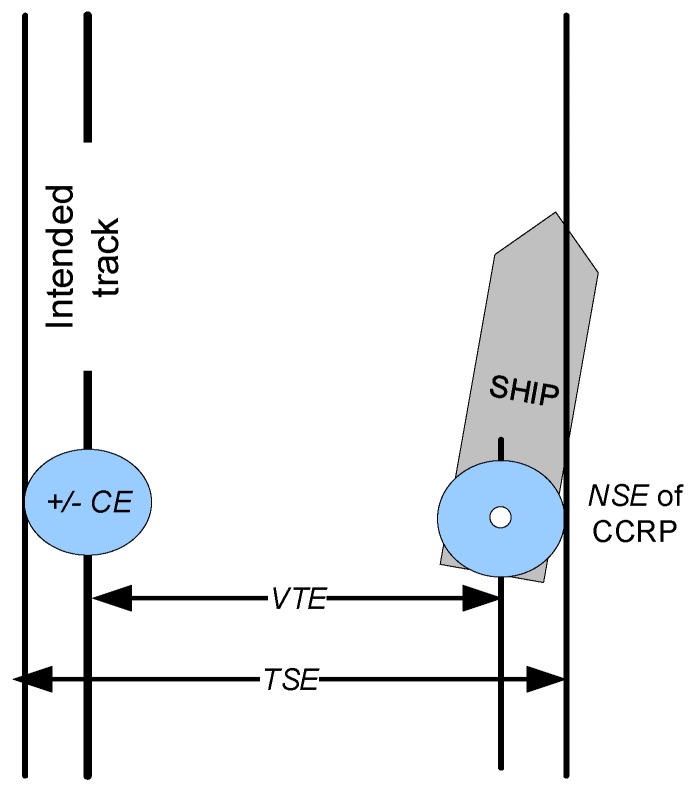
Contribution of navigation system error (*NSE*), chart error (*CE*), and vessel technical error (*VTE*) to total system error (*TSE*).

The geometric interpretation of (2) is presented in the Figure 1.

There were no additional explanations given to formulas (1) and (2), so for estimation purposes (1) should be treated conservatively as a sum of absolute values, or assuming random contributions from *CE* and *PE,* it should be transformed to the form of (2).

A.915(22) also provided more detailed performance specifications to GNSS onboard equipment (service level parameters), but only as recommendations:

- 10 m accuracy (95%) and 25 m *AL* for most applications.
- 10 s *TAL*.
- 10^−5^ *IR* per 3h.
- 99.97% continuity over 3 h.
- 99.8% overall availability (considered per 30 days).

Concluding, A.915(22) gave grounds for the application of GNSS integrity concept in the maritime domain. Its definition of reliability of position fix was a precursor to the definition of circular protection level (*PL*) that was concurrently developed by ICAO and RTCA for aviation domain [4] as an upper confidence bound on the error in the position. In order to determine *PL* value, the 1*σ* circular bound on the error in the position is derived from augmentation data (assuming multivariate normal distribution) and multiplier of this bound (further called *k*-factor) is derived from the probability of fault-free missed detection [1,3,17].

**Figure 2:**
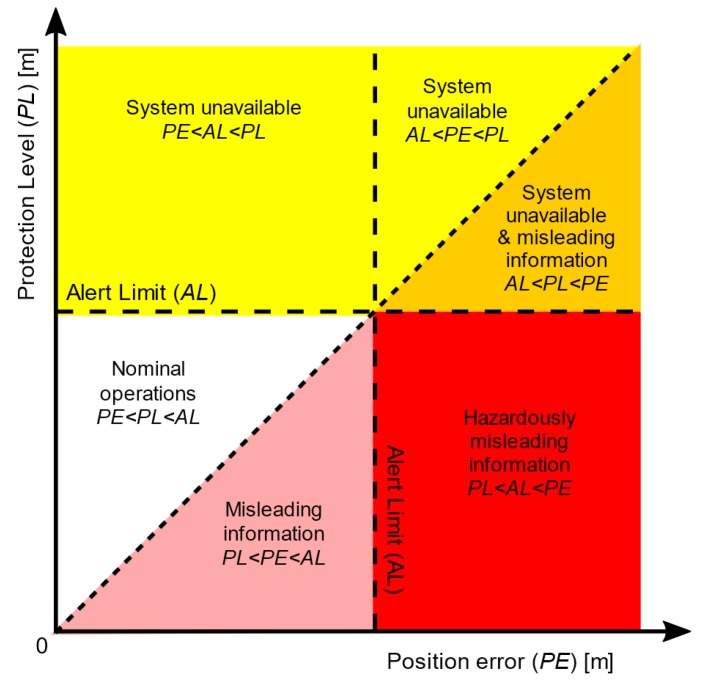
Interpretation of Stanford diagram based on 4 relations of system availability [4].

The relations among *PL*, *AL*, and *PE* could be interpreted in the Stanford diagram [4] (see the Figure 2). However, the resolution A.915(22) has not addressed the issue of the *PL* algorithm for *IR* monitoring.

In 2006 the resolution MSC.233(82) [18] on performance standards for shipborne Galileo receiver equipment was adopted. According to this resolution the Galileo shipborne receiver equipment should indicate whether the performance of Galileo is outside the bounds of requirements for general navigation in the ocean, coastal, port approach and restricted waters, and inland waterway phases of the voyage as specified in either resolution A.953(23) or Appendix 2 to resolution A.915(22) and any subsequent amendments as appropriate. The receiver equipment should as a minimum:

- Provide a warning within 5 s of loss of position or if a new position based on the information provided by the Galileo constellation has not been calculated for more than 1 s for conventional craft and 0.5 s for high-speed craft. Under such conditions the last known position and the time of last valid fix, with the explicit indication of the state so that no ambiguity can exist, should be output until normal operation is resumed.
- Use RAIM to provide integrity performance appropriate to the operation being undertaken.
- Provide a self-test function.
- For receivers having the capability to process the Galileo Safety of Life Service, integrity monitoring and alerting algorithms should be based on a suitable combination of the Galileo integrity message and RAIM. The receiver should provide an alarm within 10 s *TTA* of the start of an event if an *AL* of 25 m Horizontal Alert Limit (*HAL*) is exceeded for a period of at least 3 s. The probability of detection of the event should be better that 99.999% over a 3 h period (integrity risk <= 10^−5^/3 h).

This resolution set the first standards of GNSS subsystem based on A.915(22) and it has gone a step ahead of GPS and GLONASS performance standards. Nevertheless, it has left the problem of integrity monitoring and alerting algorithms unresolved. Even though in the norm IEC 61108-3:2010 based on MSC.233(82) was issued 2010, no maritime-specific Galileo receivers have been manufactured until now [19].

In 2007 the resolution MSC.252(83) [20] on revised performance standards for integrated navigation systems (INS) was adopted. This resolution stipulates that integrity of information should be checked by comparison of the data derived independently from at least two sensors or sources—if available—and an approved back-up should be available for the following INS sensors and sources: EPFS; heading measurement; speed measurement; radar; chart database. Data which does not pass the plausibility and validity checks with a positive result should not be used by the INS and should not affect functions that are not dependent on these data, unless the relevant performance standards specifically allow use of invalid data.

In 2011 the resolution A.1046(27) on worldwide radionavigation system [21] was adopted. It revoked previous resolution A.953(23) on IMO policy on the recognition and acceptance of suitable radionavigation systems intended for international use. A.1046(27) contains system-level specifications and has not addressed any integrity algorithms. It also has not referred to A.915(22) explicitly but has implicitly changed the previously recommended 3 h continuity time-range to 15 min and 30 days availability time-range to an indefinite value.

In 2014 the resolution MSC.379(93) [22] on performance standards for shipborne Beidou receiver equipment was adopted. It follows provisions of MSC.233(82) for European Galileo with the exception of safety of life service, which is not provided by Beidou presently.

In 2015 the resolution MSC.401(95) [23] on performance standards for multi-system shipborne radionavigation receivers was adopted. Its aim is to ensure that ships are provided with resilient position-fixing equipment suitable for use with available radionavigation systems throughout their voyage. This resolution generalizes integrity monitoring again by stipulating that the radionavigation equipment should be designed to provide means of integrity monitoring for each position, velocity, timing (PVT) source employed (e.g., RAIM, CAIM); and multi-source autonomous integrity monitoring (envisioned to be a cross-check between independent PVT sources). Later, in 2017, this resolution was amended by MSC.432(98) [24]. The amendment was short but meaningful—referring performance standards to the resolutions on stand-alone shipborne radionavigation receivers: “Type-specific performance standards for stand-alone shipborne radionavigation receivers should be taken into account when conducting type approval for multi-system receivers in accordance with resolution MSC.401(95).” Nevertheless, the MSC 401(95) enabled the full use of relevant data originating from current and future radionavigation services; thus, it allowed SBAS augmentation data processing.

In 2017 Circular MSC.1/Circ.1575 [5] on Guidelines for Shipborne Position, Navigation and Timing Data Processing (PNT DP) was adopted. This circular recommends how PNT integrity should be monitored in the maritime PNT equipment. Firstly, methods and thresholds used by the PNT DP for integrity monitoring should be qualified to evaluate if the supported accuracy level of PNT output data has been achieved or not. Therefore, the accuracy level is proposed as intra-system *AL* or threshold value to differ between fulfilled and failed requirements on PNT data output. Secondly, the *TTA* should be the tolerated time span for accuracy evaluation by the PNT DP. Thirdly, it is recommended to manufacturers to predetermine the *IR* of the applied integrity monitoring methods, taking into account application-relevant time periods under nominal conditions, if practicable. If the PNT-DP supports a redundant provision of PNT and integrity data in relation to the same accuracy level, the *IR* should be pre-evaluated for application-relevant time periods and provided as configuration parameter to ensure that the most reliable PNT data are selected for output. MSC.1/Circ.1575 also proposed some concepts of:

(1) Consistency tests using two sensors or model of ship’s movement.
(2) Determination of PL by RAIM.

For example six position solutionsh can be determined with the five consistent ranges: the all-in-view solution (Pos_AIV_) and the solutions achieved with any set of five ranges. The position error per solution depends on the expected standard deviation of position error and a *k* expansion factor. The largest distance of an estimated position error (for example *σ*_4_ of the 4th position solution Pos_4_) to the Pos_AIV_ is determined as protection level:

(3) $$PL=\left|Pos_{4}-Pos_{AIV}\right|+k\sigma_{4}.$$

MSC.1/Circ.1575 has addressed the RAIM integrity concept (without delving into probability of different fault detections as elaborated in [25,26,27]) but not the SBAS one. SBAS, and in particular EGNOS capability, is already present in some shipborne receivers. Nevertheless, currently there are neither mature standards nor regulations to define how the vessel has to process SBAS data and in particular to use SBAS for integrity purposes. Non SOLAS (IMO Convention on Safety of Life at Sea, 1974) compliant SBAS-enabled receivers are not standardised, and they make use of SBAS open services with no guarantees. SOLAS ships should not rely on SBAS messages for either accuracy or integrity until the SBAS performance for maritime receivers is properly standardised.

In 2018 DNV GL released autonomous and remotely operated ship guidelines [28]. In appendix D of these guidelines the only stipulation, additional to current IMO regulations regarding EPFS is: “minimum two separate and independent EPFS based on different technologies, both suitable for the area of operations should be part of the grounding avoidance system.” 

The questions that are still valid are: (1) Is that enough? (2) What EPFS integrity metrics should be used?

## 4. SBAS-Based Maritime Vessel Protection Area Concept

In 2017, a Maritime Vessel Protection Area (MVPA) concept was introduced by the info note to IMO Navigation, Communication, Search and Rescue Subcommittee [1]. The MVPA was developed from the Horizontal Protection Level (*HPL*) model defined within the Appendices A and J of the Minimum Operational Performance Standards (MOPS) for airborne equipment [4]. It was based on the broadcast of differential GPS corrections in message types MT1-5,7,9,17-18,24-26 and corresponding integrity data in MT2-6,10,24,26-28 by EGNOS geostationary satellites (PRN120, PRN124, and PRN126). In the present research such a concept has been adopted as well. The input quantities for the GNSS (systems other than GPS will be augmented by EGNOS in near future) integrity algorithm on the user side have been assumed as:

(1) Geometry data of GNSS satellites in the form of geometry matrix G of size *n* × 4:
  (4) $$G=\left[\begin{array}{cccc} -\cos el_{1}\sin Az_{1} & -\cos el_{1}\cos Az_{1} & -\sin el_{1} & 1\\ -\cos el_{2}\sin Az_{2} & -\cos el_{2}\cos Az_{2} & -\sin el_{2} & 1\\ \cdots & \cdots & \cdots & \cdots \\ -\cos el_{n}\sin Az_{n} & -\cos el_{n}\cos Az_{n} & -\sin el_{n} & 1 \end{array}\right],$$
  where *el_i_* and *Az_i_* are the elevation and azimuth angles between the receiver antenna and the *i*th satellite (*i* = 1,2,...,*n*), and *n* is the number of visible satellites, respectively.
(2) Estimated user differential range error *σ_i_*_,UDRE_ [m] based on UDREI*_i_* indicator whose components are transmitted in EGNOS messages MT2-6,24 (section A.4.4.4 of [4]).
(3) Estimated grid ionospheric vertical error *σ_i_*_,GIVE_ [m] based on GIVEI*_i_* indicator transmitted in EGNOS message MT26 (section A.4.4.10 of [4]).
(4) Residual tropospheric error parameter *σ_i_*_,*tropo*_ [m], calculated according to the model defined within sections A.4.2.4 and A.4.2.5 of [4]:
  (5) $$\sigma_{i,tropo}=0.12\frac{1.001}{\sqrt{0.002001+\sin^{2}el_{i}}}.$$
(5) Estimated error of shipborne receiver *σ_i,mr_* [m], depending on receiver properties, derived by analogy to the model defined within section J.2.4 of [4]:
  (6) $$\sigma_{i,mr}=\left(\sqrt{\sigma_{i,noise}^{2}+\sigma_{i,multipath}^{2}+\sigma_{i,divg}^{2}}\right)\;,$$
  where:
  *σ_i_*_,*multipath*_ is the estimated multipath error [m] depending on *i*th satellite elevation *el_i_*; the receiver’s properties, and site-specific GNSS signal reflections, which must be locally evaluated (this alone variance cannot be derived from the SBAS message). The airborne reference model of this error can be found in [4] but a universal concept of multipath model in marine environment is still to be developed;
  *σ_i_*_,*divg*_ is estimated error [m] induced by the steady-state effects (divergence) of shipborne receiver smoothing filter assumed to be equivalent to the one presented in [4];
  *σ_i_*_,*noise*_ is estimated error [m] associated with GPS receiver for *i*th satellite, including receiver noise, thermal noise, interference, inter-channel biases, extrapolation, time since smoothing filter initialization, and processing errors; assumed to be equivalent to the one presented in [4];

On the basis of the input quantities the weight matrix *W* is built under assumption of uncorrelated, EGNOS corrected measurements characterized by the inverse variances of the distances to the observed satellites. These variances are calculated according to:

(7) $$\sigma_{i}^{2}=\sigma_{i,flt}^{2}+\sigma_{i,UIRE}^{2}+\sigma_{i,tropo}^{2}+\sigma_{i,mr}^{2},$$

(8) $$W=\left[\begin{array}{cccc} \frac{1}{\sigma_{1}^{2}} & 0 & \cdots & 0\\ 0 & \frac{1}{\sigma_{2}^{2}} & \cdots & 0\\ \vdots & \vdots & \ddots & \vdots \\ 0 & 0 & \cdots & \frac{1}{\sigma_{n}^{2}} \end{array}\right],$$

where, in (7):

*σ_i_*_,*flt*_^2^ is the model variance for the residual error associated to *σ_i_*_,UDRE,_ as defined in section A.4.5.1 of [4] [m^2^],
*σ_i_*_,*UIRE*_^2^ is the model variance for the slant range ionospheric error associated to *σ_i_*_,GIVE,_ as defined in sections A.4.4.10 and A.4.5.2 of [4] [m^2^],

Finally, the point positioning covariance matrix is found:

(9) $$C=\left[\begin{array}{cccc} s_{E}^{2} & s_{EN} & s_{EU} & s_{ET}\\ s_{EN} & s_{N}^{2} & s_{NU} & s_{NT}\\ s_{EU} & s_{NU} & s_{U}^{2} & s_{UT}\\ s_{ET} & s_{NT} & s_{UT} & s_{T}^{2} \end{array}\right]={\left(G^{T}WG\right)}^{-1},$$

where:

*s_E_*^2^ is the variance of the antenna receiver Easting measurement in the local reference frame centred on the GPS antenna (East, North, Up, ENU) [m^2^];
*s_N_*^2^ is the variance of antenna receiver Northing measurement in the local reference frame (ENU) [m^2^];
*s_U_*^2^ is the variance of antenna receiver vertical measurement [m^2^];
*s_T_*^2^ is the variance of receiver time correction measurement multiplied by speed of light [m^2^];

and, finally, the mixed terms (e.g., *s_EN_*, etc.) are the co-variances of respective measurements [m^2^].

A “circular” assessment of the user positioning integrity is derived as the *HPL*:

(10) $$HPL=k\sqrt{\frac{s_{E}^{2}+s_{N}^{2}}{2}+\sqrt{{\left(\frac{s_{E}^{2}-s_{N}^{2}}{2}\right)}^{2}+s_{EN}^{2}},}$$

The rationale for the *k* coverage factor (or elliptical scale factor) in (10) comes from the assumption of uncertainty normal distributions in both the North and the East directions of position parameters and assumption of integrity risk value defined as the probability that the user will experience a true position outside the *HPL* and possibly *AL* without being informed within the *TTA*. In the simulation study described in the following sections of this paper the *k* for GNSS maritime equipment has been calculated according to (17) to achieve integrity risk value specific for the operation or area set in [3]. A method developed in [29] has been taken into account as well. In comparison the *k* for GNSS airborne equipment has been set by RTCA and adopted by ICAO as 6.18 for en-route lateral navigation (LNAV) and 6.0 for navigation with vertical guidance [4]. 

**Figure 3:**
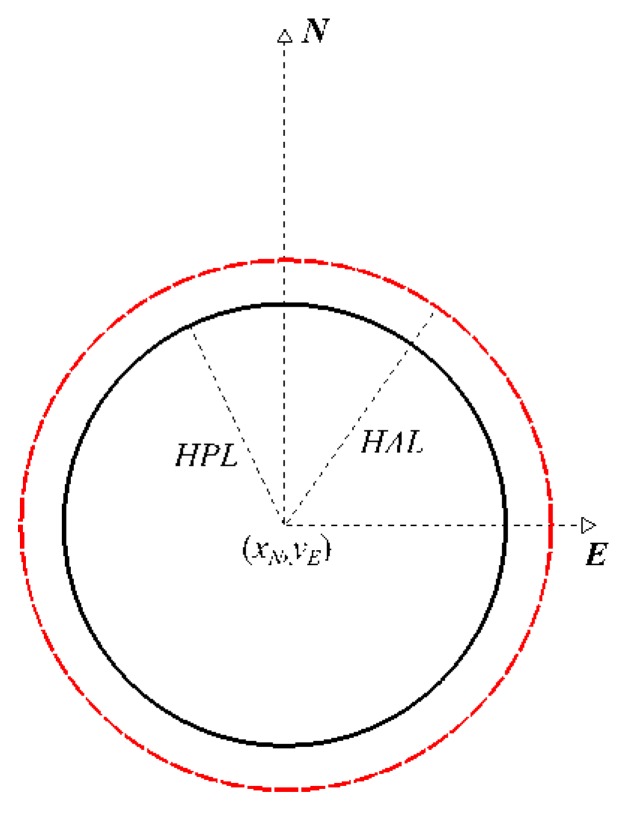
Horizontal protection level and alert limit.

Such a “circular’ *HPL* of fixed confidence (10) is compared with *HAL* as shown in Figure 3.

**Figure 4:**
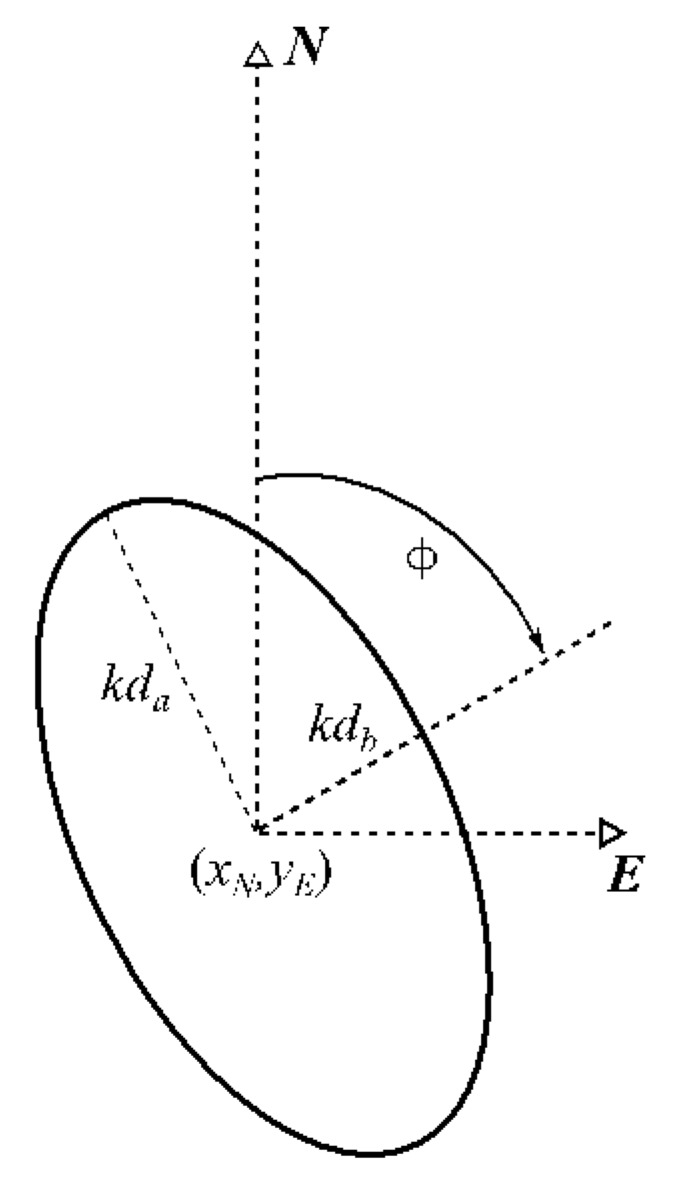
Elliptical representation of an SBAS based protection area.

The algorithm of the GNSS positioning integrity assessment in maritime domain has been further developed by inclusion of a protection ellipse (*PE_mr_*), which is specified by 4 parameters, i.e.,: (1) semi-major axis of the estimated position error ellipse *d_a_* [m], (2) semi-minor axis of the error ellipse *d_b_* [m], (3) orientation of the error ellipse *Φ*, and (4) coverage factor *k* (see Figure 4).

(11) $$PE=\left\{\begin{array}{c} kd_{a}\\ kd_{b}\\ \Phi \end{array}.\right.$$

where:

(12) $$d_{a}=\sqrt{\frac{s_{E}^{2}+s_{N}^{2}}{2}+\sqrt{{\left(\frac{s_{E}^{2}-s_{N}^{2}}{2}\right)}^{2}+s_{EN}^{2}}},$$

(13) $$d_{b}=\sqrt{\frac{s_{E}^{2}+s_{N}^{2}}{2}-\sqrt{{\left(\frac{s_{E}^{2}-s_{N}^{2}}{2}\right)}^{2}+s_{EN}^{2}}},$$

(14) $$\Phi =\frac{\pi }{2}-\frac{1}{2}\mathrm{atan}2\left(2s_{EN}^{},s_{E}^{2}-s_{N}^{2}\right),$$

where *Φ* is a clockwise angle of rotation from North either of the semi-major ellipse’s axis (if *s_E_* > *s_N_*) or of the semi-minor axis (if *s_N_* > *s_E_*); and

atan2 is the directed angle arctangent function of arguments (*y*, *x*) in Cartesian reference frame.

The parameters of formulae (12), (13), and (14) are derived from the square root of eigenvalues of the covariance matrix (9) confined to:

(15) $$C_{PA}=\left[\begin{array}{cc} s_{E}^{2} & s_{EN}\\ s_{EN} & s_{N}^{2} \end{array}\right].$$

(14) is the direction of the eigenvector of (15).

The probability that the user will experience a true position inside the protection ellipse is conservatively calculated using the addition theorem for the chi-square distribution:

(16) $$p=1-e^{\frac{-k^{2}}{2}},$$

and the scale factor:

(17) $$k=\sqrt{\left(-2\ln(1-p\right)}.$$

For example, to create a 95% confidence *HPL* from the 1*σ* error circle a factor of *k* ≈ 2.45 (17) should be used; to get a 99.8% confidence *HPL* a factor of *k* ≈ 3.53 should be used, and so on. If error-correlation time of 150 s is assumed and treated as integrity epoch correspondingly to aviation standards [4], then a 3 h operation interval recommended by IMO in resolution A.915 [3] will contain 3 × 3600 s/150 s = 72 statistically independent epochs. This gives per-epoch integrity risk probability of:

(18) $$IR=\frac{10^{-5}}{72}\approx 1.39\times 10^{-7}.$$

*IR* can be relaxed for 15-minute operation interval, as proposed in [21]:

(19) $$IR=\frac{150\times 10^{-5}}{15\times 60}\approx 1.67\times 10^{-6}.$$

It can also be treated conservatively, assuming an error correlation time equivalent to a position update time of 2 s [21] and critical operation time of 3 h:

(20) $$IR=\frac{2\times 10^{-5}}{3\times 3600}\approx 1.85\times 10^{-9}.$$

An initial assessment the *k* factor of 5.62 has been suggested. This corresponds to the integrity risk given by (18).

The “elliptical” presentation of a protection area provides navigator with the extra benefit coming from knowledge of N-E variances and their covariance resulting in changes of the ellipse’s orientation and shape. That is why the concept of MVPA has been further developed for Electronic Chart Data Information System (ECDIS) where a vessel is shown as a 2-dimensional spatial object (a model of ship’s hull contour) [17]. The detailed mathematical model is as follows.

A geometry of the vessel’s contour in the ECDIS display is expressed by the following two observation equations:

(21) $$x_{j,N}=x_{N}-x_{GPS}+d_{j}\cos\left(\psi +\alpha_{j}\right),$$

(22) $$y_{j,E}=y_{E}-y_{GPS}+d_{j}\sin\left(\psi +\alpha_{j}\right),$$

where:

(23) $$d_{j}=\sqrt{{x_{j}}^{2}+{y_{j}}^{2}},$$

(24) $$\alpha_{j}=\frac{\pi }{2}-\mathrm{atan}2\left(x_{j},y_{j}\right),$$

**Figure 5:**
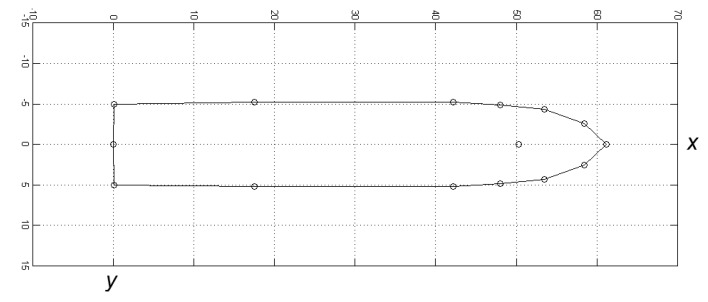
Model vessel’s contour consisting of 14 points in the body-fixed reference metric frame and GNSS antenna’s position in the fore part.

*x_j_, y_j_* are the calculated coordinates of consecutive *j* points of ship’s contour in the body-fixed reference frame (this is fixed to the vessel at the common reference point of aft perpendicular with positive *x* axis to fore, *y* axis to starboard, following the convention used in maritime craft hydrodynamics - (see Figure 5);
*x_GPS_, y_GPS_* are the coordinates (offsets from 0 at aft perpendicular) of EGNOS augmented GPS receiver antenna in the body-fixed reference frame;
*x_j,N_*, *y_j,E_* are the calculated coordinates of consecutive *j* points of ship’s contour in the local reference frame (ENU);
*x_N_*, *y_E_* are the recorded positions of EGNOS augmented GPS receiver antenna in the local reference frame (ENU);
*ψ* is the heading of vessel counted clockwise from North in the local reference frame (ENU);
*d_j_* is the *j*th distance between GPS antenna and *j*th point of ship’s contour; and
*α_j_* is the *j*th angle between GPS antenna and *j*th point of ship’s contour counted clockwise from *x-*axis in the body-fixed reference frame.

The errors of data in Equations (21) and (22) propagate into the final MVPA according to the Gauss’s Error Propagation Law. The systematic errors of *x_GPS_*, *y_GPS_*, *d_j_*, and *α_j_* can be minimized to a negligible magnitude by a precise dimensional control. Therefore, only the propagation of other parameters’ errors (*x_N_*, *y_E_*, *ψ*) is taken into account in the MVPA determination according to the formula:

(25) $$C_{j,PA}=J_{j}C_{O}{J_{j}}^{T},$$

where:

*C_j,PA_* is the covariance matrix of derived quantities:
  (26) $$C_{j,PA}=\left[\begin{array}{cc} s_{j,E}^{2} & s_{j,EN}\\ s_{j,EN} & s_{j,N}^{2} \end{array}\right],$$
*s^2^_j,E_* is the Easting variance of consecutive *j* points of ship’s contour in the local reference frame (ENU) [m^2^];
*s^2^_j,N_* is the Northing variance of consecutive *j* points of ship’s contour in the local reference frame (ENU) [m^2^];
*s_j,EN_* is the covariance of *j* points respective coordinates [m^2^];
*J_j_* is the Jacobian matrix (matrix of all first-order partial derivatives) of Equations (21) and (22), excluding *x_GPS_*, *y_GPS_* due to their negligible errors:
  (27) $$J_{j}=\left[\begin{array}{cccc} 1 & 0 & \sin(\psi +\alpha_{j}) & d_{j}\cos\left(\psi +\alpha_{j}\right)\\ 0 & 1 & \cos(\psi +\alpha_{j}) & -d_{j}\sin\left(\psi +\alpha_{j}\right) \end{array}\right].$$
*C_O_* is the covariance matrix of observations:
  (28) $$C_{O}=\left[\begin{array}{cccc} s_{E}^{2} & s_{EN} & 0 & 0\\ s_{EN} & s_{N}^{2} & 0 & 0\\ 0 & 0 & 0 & 0\\ 0 & 0 & 0 & s_{\psi }^{2} \end{array}\right],\;$$

where:

*s*^2^*_ψ_* is the marine vessel heading variance, relevant to the marine-specific attitude/heading equipment (the typical values for marine gyros in transport vessels are in range 0.5–1°); and:
*J_j_^T^* is the transposed Jacobian matrix (27).

Estimated error of each *j*th ship’s contour point involves the errors of two jointly distributed variables of *x_j,N_* and *y_j,E_* coordinates. Thus, the positional error follows a bivariate normal distribution. Taking above into account, to fully describe the estimated error of each *j*th point, it is necessary to determine the orientation *Φ_j_* and lengths of the semi-major *d_j,a_* and semi-minor axes *d_j,b_* of the *j*th error ellipses according to the formulas analogical to (12)–(14):

(29) $$d_{j,a}=\sqrt{\frac{s_{j,E}^{2}+s_{j,N}^{2}}{2}+\sqrt{{\left(\frac{s_{j,E}^{2}-s_{j,N}^{2}}{2}\right)}^{2}+s_{j,EN}^{2}}},$$

(30) $$d_{j,b}=\sqrt{\frac{s_{j,E}^{2}+s_{j,N}^{2}}{2}-\sqrt{{\left(\frac{s_{j,E}^{2}-s_{j,N}^{2}}{2}\right)}^{2}+s_{j,EN}^{2}}},$$

(31) $$\Phi_{j}=\frac{\pi }{2}-\frac{1}{2}\left(\mathrm{atan}2\left(2s_{j,EN}^{},s_{j,E}^{2}-s_{j,N}^{2}\right)+\psi \right),$$

where *Φ_j_* is a clockwise angle of rotation from the ship’s body-fixed *x-*axis either of the semi-major ellipse’s axis (if *s_j,E_*>*s_j,N_*) or the semi-minor axis (if *s_j,E_*<*s_j,N_*).

Each of the determined *j* ellipses are further enlarged to the established confidence level by multiplying *d_j,a_* and *d_j,b_* by a coverage factor *k* (17).

**Figure 6:**
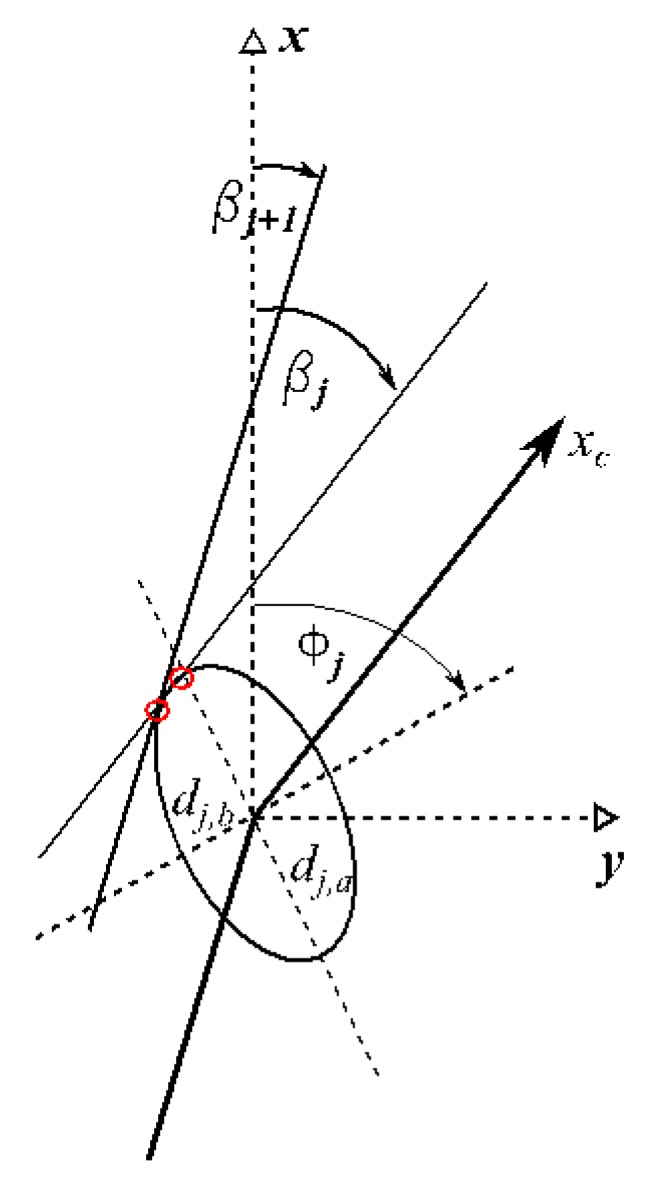
Construction of two tangent points to the *j*th error ellipse.

Knowing the parameters of uncertainty ellipses (29)–(31) centred in *j* points of ship’s contour the next step is to find the extreme outer points of these ellipses in order to construct the boundary of MVPA (Figure 6).

The algorithm is as follows:

The angle *β_j_* of the line leading through *j* and *j*+1 points (*j* = 1 is set as *j*+1 in case of max. *j* achieved) counted clockwise from *x-*axis in body-fixed reference frame are determined according to the formula:

(32) $$\beta_{j}=\frac{\pi }{2}-\mathrm{atan}2\left(x_{j}-x_{j+1},y_{j}-y_{j+1}\right).\;$$

Tangent points of ellipses with lines of slope *β_j_* are determined according to the formulas:

(33) $$\Phi_{j,c}=\frac{\pi }{2}-\Phi_{j}+\beta_{j},$$

(34) $$R_{j}=\left[\begin{array}{cc} \cos\beta_{j} & -\sin\beta_{j}\\ \sin\beta_{j} & \cos\beta_{j} \end{array}\right],$$

(35) $$A_{j,1}=d_{j,b}^{2}\sin^{2}\Phi_{j,c}+d_{j,a}^{2}\cos^{2}\Phi_{j,c},$$

(36) $$A_{j,2}=d_{j,b}^{2}\cos^{2}\Phi_{j,c}+d_{j,a}^{2}\sin^{2}\Phi_{j,c},$$

(37) $$t_{j,1}=\sqrt{\frac{-A_{j,1}d_{j,a}^{2}d_{j,b}^{2}}{\cos^{2}\Phi_{j,c}\sin^{2}\Phi_{j,c}{\left(d_{j,b}^{2}-d_{j,a}^{2}\right)}^{2}-A_{j,1}A_{j,2}}},$$

(38) $$T_{j,2}=\left[\begin{array}{cc} t_{j,1} & \frac{-t_{j,1}\cos^{2}\Phi_{j,c}\sin^{2}\Phi_{j,c}\left(d_{j,b}^{2}-d_{j,a}^{2}\right)}{A_{j,1}}\\ -t_{j,1} & \frac{t_{j,1}\cos^{2}\Phi_{j,c}\sin^{2}\Phi_{j,c}\left(d_{j,b}^{2}-d_{j,a}^{2}\right)}{A_{j,1}} \end{array}\right],$$

(39) $$T_{j,3}=T_{j,2}R,$$

(40) $$\left[\begin{array}{cc} y_{j,tp} & x_{j,tp}\\ y_{j,tn} & x_{j,tn} \end{array}\right]=\left[\begin{array}{cc} T_{j,3}\left(1,1\right)+y_{j} & T_{j,3}\left(1,2\right)+x_{j}\\ T_{j,3}\left(2,1\right)+y_{j} & T_{j,3}\left(2,2\right)+x_{j} \end{array}\right],$$

where:

*Φ_j,c_* is the counter-clockwise angle of the *j*th ellipse rotation to *x*-axis in standard Cartesian 0*xy* reference frame,
*x_j,tp_, y_j,tp,_ x_j,tn_, y_j,tn_* are the coordinates [m] of consecutive *j* tangent points in body-fixed reference frame, the extreme outer points are either *x_j,tp_, y_j,tp_* if *β_j_* > 0 or *x_j,tn_, y_j,tn_* if *β_j_* ≤ 0.

**Figure 7:**
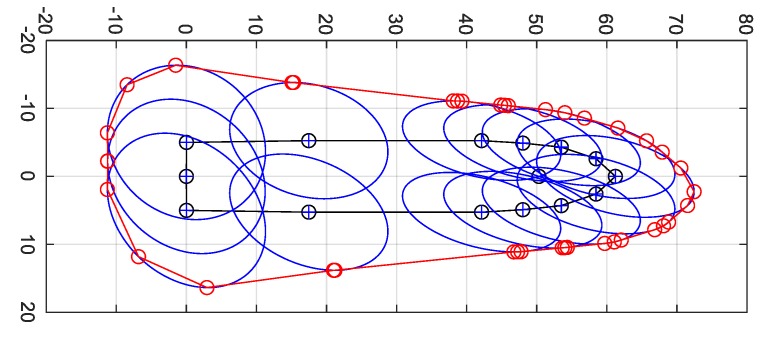
Example of MVPA visualization [m] for 2° heading error of m/v “Nawigator XXI”

The MVPA is constructed by linear connection of the resultant tangent points as in Figure 7. This way the boundary spline representing the furthest points of ellipses in respect to the ship’s hull is found. In order to minimize the linear spline approximation error, the number of tangent points can be increased adding extra tangent lines of slope angles in the range between *β_j_* and *β_j_*_+1_ (see Figure 6).

The example of MVPA presented in Figure 7 was based on the following construction data :

- Vessel dimensions and 2D contour: derived from shipyard data of m/v “Nawigator XXI”, MMSI 261187000; LOA = 60.21 m, *B* = 10.5 m.
- Heading: *ψ* = 45°, heading error estimate: *s_ψ_* = 2° (1σ), EGNOS based error estimates of common reference point position of GNSS antenna in fore part: *s_E_* = 1 m, *s_N_* = 2 m, *s_EN_* = 0.8 m^2^; integrity risk expansion factor *k* = 5.67.

In addition to MVPA construction, the most conservative value of *HPL* is calculated taking into account ship’s heading and its uncertainty based on (25), (26), and (29):

(41) $$HPL_{MVPA}=\max d_{j,a}=\max\left(k\sqrt{\frac{s_{j,E}^{2}+s_{j,N}^{2}}{2}+\sqrt{{\left(\frac{s_{j,E}^{2}-s_{j,N}^{2}}{2}\right)}^{2}+s_{j,EN}^{2}}}\right).$$

Example calculation of *HPL* based solely on GNSS (10) and *HPL_MVPA_* (41) for various values of heading error estimations *s_ψ_* and data as used in Figure 7 is shown in Table 1.

## 5. Alert Limit Decision Model–Operational Shipborne Position Sensors’ Integrity Concept

The proposed shipborne position sensors’ integrity concept uses both adaptive safety margins and navigation warning lights concept. Taking this into account, the proposed models are based on the following two assumptions:

(1) Protection levels are calculated over time period up to some probability, thus the vessel’s body position (unknown true horizontal position of ship’s hull whose parameters are latitude, longitude and true heading) is located in an area with a certain confidence (integrity risk). This safety region (MVPA) could provide key support information to the navigator or captain to perform safer operations.
(2) *VTE* estimations and resultant safe manoeuvring area are calculated for specific hydro-meteorological conditions (or their safety-critical limits) on the basis of knowledge of historical statistics of various ships’ motion (mean and standard deviation of ship’s contour distance from the set route segment or a centre of the fairway coming from simulation studies, automatic identification system AIS or analytical models) in relation to the available water area (safety isobaths or their tangent approximations constructing a virtual adaptive approach channel VAAC).

**Figure 8:**
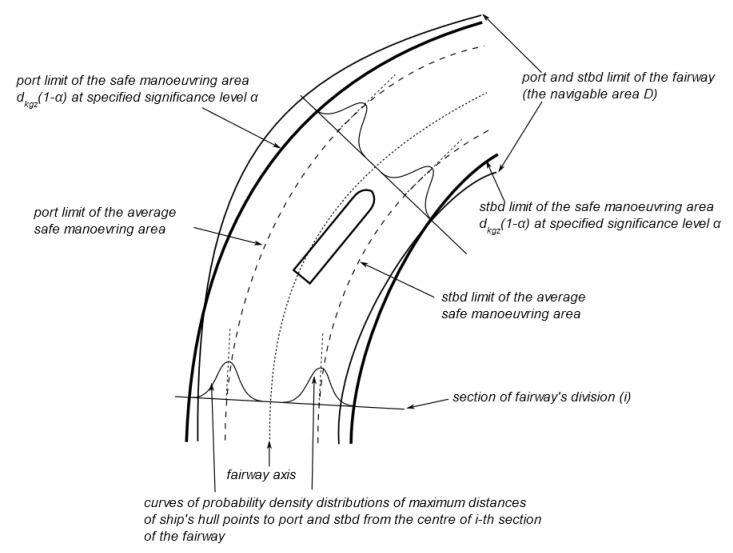
Construction of the safe manoeuvring area or *VTE* limits.

The methodology of maritime navigation channels parameters determination, which could be directly followed in order to estimate *VTE* boundary of MASS (either steered remotely or via autopilot), was presented in [30] (Figure 8).

Monitoring of ship’s position in relation to assumed *VTE* limit (VTEL) can be treated as verification of ship’s proper manoeuvring behaviour. The ship that crosses VTEL misbehaves in terms of conformance to the statistically significant majority of manoeuvres. Since MVPA bound based on GNSS *HPL* evolves over time, it can be also seen as an adaptive safety margin concept if assessed in relation to assumed VTEL and VAAC (*AL*). Additionally the system can raise an alarm when the navigation system is providing a solution whose position error bound exceeds certain thresholds (*AL*) and compromises the safety of ship’s operation. The idea of this is that certain alarms/warnings can be triggered either in relation with different *AL* or service levels linked with IMO A.915(22) or adaptively as *AL* changes during route monitoring (VTEL in relation to VAAC, the distance between two never less than *AL* limits).

In order to determine the minimum and maximum distances of the MVPA to the boundary lines (VTEL and VAAC), the distance *d_Bj,k_* to the rectangular projection of the selected MVPA point on the straight line containing the *k*th section of the boundary line is determined.

(42) $$d_{Bk}=s_{d}\min d_{Bj,k}=s_{d}\min P_{j,t}P_{Bk}=s_{d}\min\sqrt{{\left(x_{Bk}-x_{j,t}\right)}^{2}+{\left(y_{Bk}-y_{j,t}\right)}^{2}}$$

where:

*d_Bk_* is the minimum distance of the MVPA to the *k*th segment of the boundary line [m],
*s_d_* is either +1 or −1 depending on directions of vectors $\vec{P_{j,t}P_{Bk}}$, $\vec{P_{j}P_{j,t}}$ and ship’s heading; if both vectors are directed to the same half side of ship’s heading line than *s_d_* is positive in other case it is negative,
*P_Bk_*(*x_Bk_*, *y_Bk_*) is the point of coordinates *x_Bk_*, *y_Bk_* [m] of the rectangular projection of the selected MVPA point on the boundary line,
*x_j,t_, y_j,t_* are the coordinates [m] of consecutive *j*th ellipse tangent points converted to a local reference frame of ENC data.

Because boundary lines are represented in the electronic navigation chart data as an array of points *Pk*(*xk*, *yk*), the coordinates of the rectangular projection point *P_Bk_*(*x_Bk_*, *y_Bk_*) are found as a result of solving the system of equations:

(43) $$\left\{\begin{array}{c} \left(y_{Bk}-y_{k}\right)\left(x_{k+1}-x_{k}\right)=\left(y_{k+1}-y_{k}\right)\left(x_{Bk}-x_{k}\right)\\ \left(y_{k+1}-y_{k}\right)\left(y_{Bk}-y_{j,t}\right)=-\left(x_{k+1}-x_{k}\right)\left(x_{Bk}-x_{j,t}\right) \end{array}\right.$$

(44) $$\begin{array}{c} x_{Bk}=\frac{\left(y_{k+1}-y_{k}\right)\left(x_{k+1}-x_{k}\right)\left(y_{j,t}-y_{k}\right)+x_{j,t}{\left(x_{k+1}-x_{k}\right)}^{2}+x_{k}{\left(y_{k+1}-y_{k}\right)}^{2}}{{\left(x_{k+1}-x_{k}\right)}^{2}+{\left(y_{k+1}-y_{k}\right)}^{2}}\\ y_{Bk}=\frac{\left(x_{k+1}-x_{k}\right)\left(y_{k+1}-y_{k}\right)\left(x_{j,t}-x_{k}\right)+y_{j,t}{\left(y_{k+1}-y_{k}\right)}^{2}+y_{k}{\left(x_{k+1}-x_{k}\right)}^{2}}{{\left(x_{k+1}-x_{k}\right)}^{2}+{\left(y_{k+1}-y_{k}\right)}^{2}} \end{array}$$

To determine whether the *P_Bk_* point found is actually contained in the *k*th segment, and not only in the straight line passing through it, the following condition is checked:

(45) $$\begin{array}{cc} \; & \left(\left(x_{Bk}<x_{k}\right)\wedge \left(x_{Bk}<x_{k+1}\right)\right)\vee \left(\left(x_{Bk}>x_{k}\right)\wedge \left(x_{Bk}>x_{k+1}\right)\right)\vee \\ \; & \vee \left(\left(y_{Bk}<y_{k}\right)\wedge \left(y_{Bk}<y_{k+1}\right)\right)\vee \left(\left(y_{Bk}>y_{k}\right)\wedge \left(y_{Bk}>y_{k+1}\right)\right) \end{array}$$

When conjunction of the alternative (44) is false, the correct *k*th section of the boundary line and *P_Bk_* have been found. Otherwise, calculations are made for the next *k* + 1 segment. The formulae (42)–(45) can be followed accordingly in case of other distances calculation (between lines of VTEL and VAAC, two VAACs etc.).

The following six situations can occur depending on *HPL_MVPA_* and *AL* values, and MVPA position in relation to VTEL and VAAC during passage of one way water channel. In all these situations *AL* is set adaptively as the lowest distance between VTEL and VAAC predicted ahead in time range up to 6min. The 6min value has been based on expert knowledge as advised in [20].

(1) Safe operation with green light: *HPL* < *AL*, MVPA inside VTEL.

**Figure 9:**
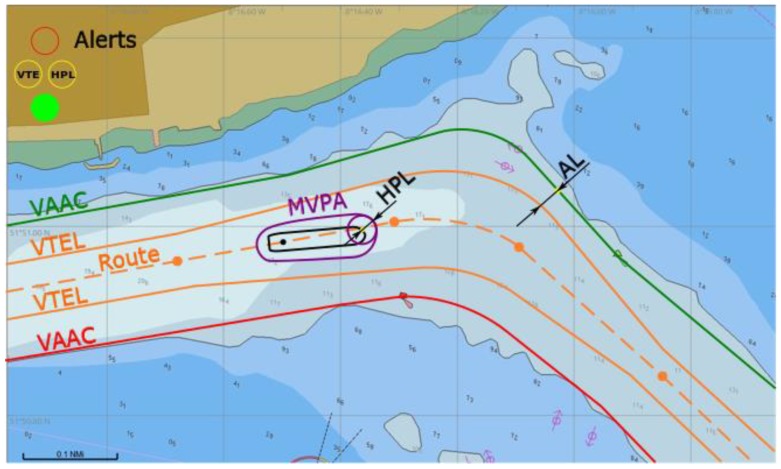
Safe operation in a one-way waterway, conceptual presentation in ECDIS.

This is a situation where a ship can safely follow her planned route (Figure 9). Both GNSS and the ship’s heading data can be trusted, and their accuracy is contained within the safety limit (no *HPL* alert), and ship’s manoeuvring / technical error is contained within safety limit (no *VTE* alert, *d_Bk_* > 0) as well.

(2) A warning is raised by yellow/orange light named HPL: *HPL* ≥ *AL.*

**Figure 10:**
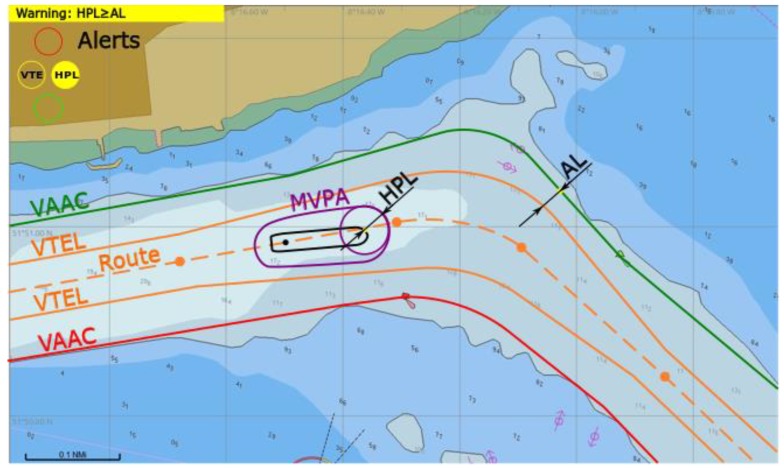
Warning of *HPL* raised, conceptual presentation in ECDIS.

There is not any *VTE* alert (*d_Bk_* > 0) but *HPL* is triggered due to its high value exceeding *AL*. A vessel should steer with utmost caution as GNSS positioning—although available—is not reliable enough (Figure 10). Other means of positioning should be used.

(3) A warning is raised by yellow/orange light named VTE: MVPA outside VTEL.

**Figure 11:**
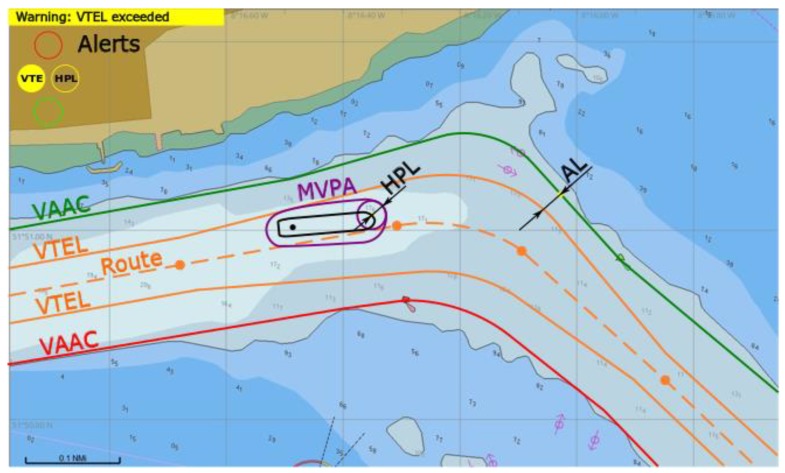
Warning of VTE raised, conceptual presentation in ECDIS.

*VTE* alert is triggered (*d_Bk_* ≤ 0) while *HPL* is within *AL*. The navigator is warned that he/she steered the vessel outside the path followed by other similar ships. He/she can expect only small safety margin coming from the *AL* value, but the situation can evolve to the one numbered 5). A vessel should steer to the route centred inside VTEL as soon as practically possible (Figure 11).

(4) An alarm is raised by a red light: *HPL* ≥ *AL*, MVPA outside VTEL.

**Figure 12:**
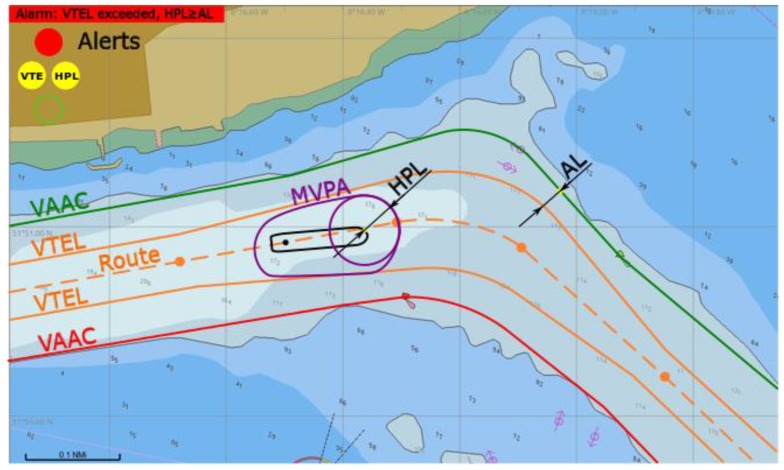
Alarm of both *VTE* and *HPL* raised, conceptual presentation in ECDIS.

Both *VTE* (*d_Bk_* ≤ 0) and *HPL* alerts are triggered. Further operation (proceeding via fairway based on GNSS) is not permitted (Figure 12). Other means of positioning should be used and then the vessel should steer back to route inside VTEL as soon as practically possible. 

(5) An alarm is raised by a red light: MVPA outside VTEL, and distance between MVPA and VTEL exceeds *AL.*

**Figure 13:**
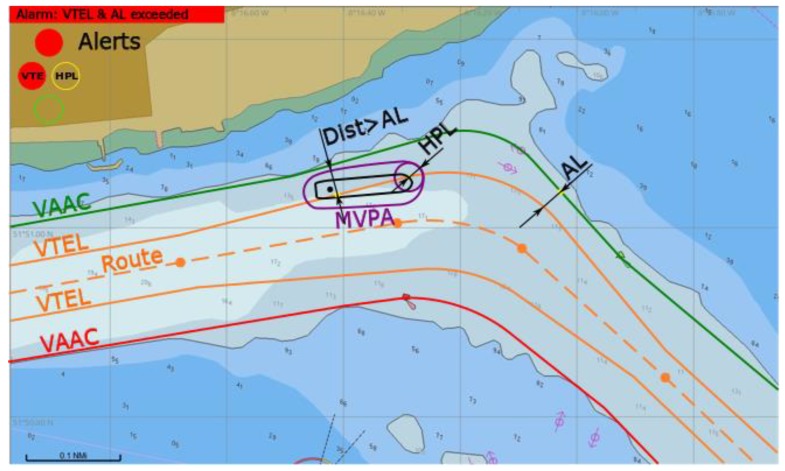
Alarm of high *VTE* raised, conceptual presentation in ECDIS.

*VTE* alert is triggered (*d_Bk_* ≤ 0) and |*d_Bk_* ≥ *AL*|. The vessel should steer back to route inside VTEL immediately as there is no safety margin left several minutes ahead. Situation can become critical in case of GNSS positioning deterioration or proceeding further without any corrective intervention (Figure 13).

(6) An alarm is raised by red light: GNSS fault, *HPL* not available.

**Figure 14:**
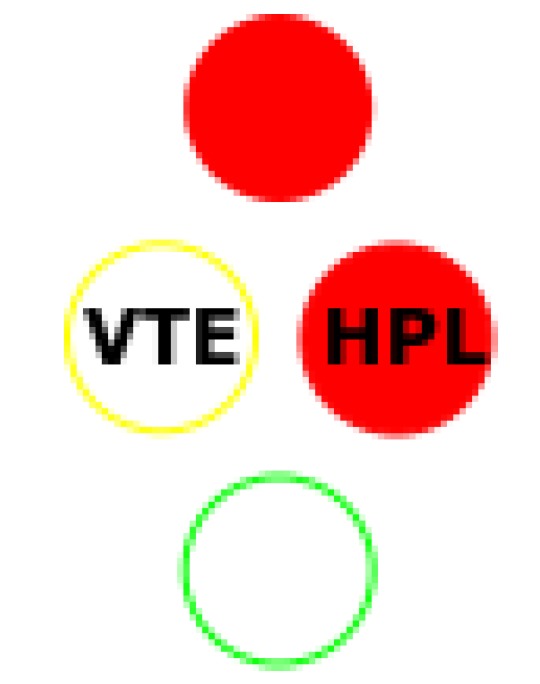
Alarm of GNSS fault—position or *HPL* not available, conceptual presentation in ECDIS.

Such a situation (Figure 14) can occur in case of GNSS signal reception problems or satellite warning flags send via GNSS or SBAS messages. Further operation with GNSS positioning is not permitted. Other means of positioning should be used.

**Figure 15:**
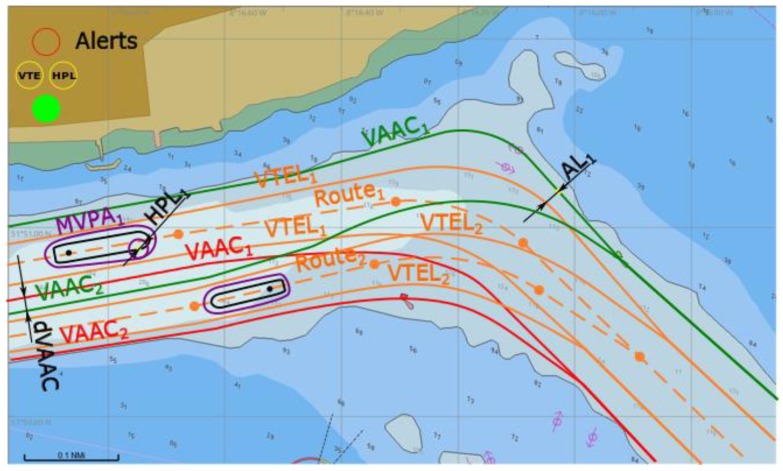
Safe operation in a two way waterway, conceptual presentation in ECDIS.

During passage of two-way water channel the proposed user integrity concept uses the adaptive safety margins and the navigation traffic lights concept in analogy to a one-way waterway, but taking into account individual VAACs and VTELs specific to each vessel (see Figure 15). For passing ships an additional safety distance is considered – minimum distance between their specific VAACs (*dVAAC*). The total available water area is equal to the sum of two VAACs widths and a minimum distance between those.

In Figure 15 situation where two passing ships can follow safely their planned routes is presented. In such a case extra distance of *dVAAC* in the waterway straight segment (designed for passing manoeuvres) is taken into account (calculated according to (41)–(44) of minimum value as advised in [30])).

## 6. Simulation Tests

Two simulation scenarios were developed in order to determine the impact of the integrity concept for MASS position sensors on the safety of navigation. 

Scenario 1: Operational passage of container vessel through a straight line section and a bend of a fairway in fixed hydrometeorological conditions. Navigation is based on standard ECDIS functionalities. Position errors are forced at the passage start: systematic directional error of position is continuously increased. Simulation of steady position drift in one direction. 

Scenario 2: Operational passage of container vessel through a straight line section and a bend of a fairway in fixed hydrometeorological conditions corresponding to scenario 1. Navigation is based on ECDIS with all position integrity functionalities enabled. Position errors are forced at the passage start: systematic directional error of position is continuously increased. Simulation of steady position drift in one direction.

**Figure 16:**
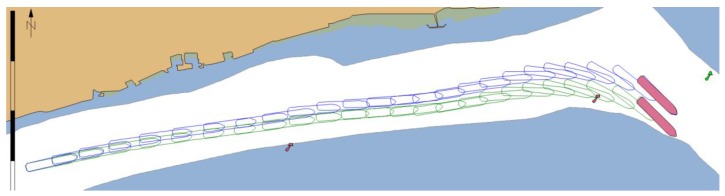

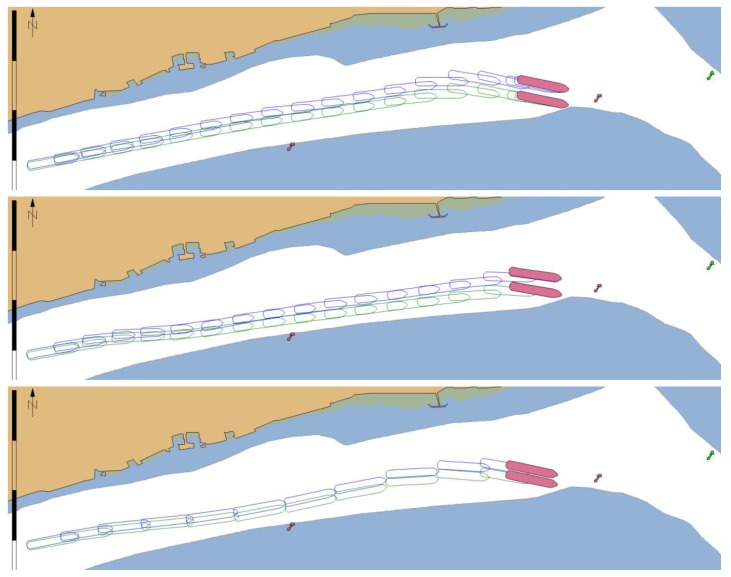
Example of 4 passages of the MASS with simulated GNSS signal deterioration – without integrity data presentation in ECDIS.

Both scenarios tested the performance of MASS remote operator/controller in group of 20 experienced navigators (master mariners and pilots) during evolving GNSS signal degradation that could be expected as a result of propagation problems in restricted water areas or even deliberate spoofing. An increasing error in north direction was simulated. Figure 16 shows the real position of the ship (green outline) and the calculated position that was presented to the navigator (blue outline). Each case resulted in ship’s grounding when system without functionalities of PNT data integrity was used (position marked in red).

**Figure 17:**
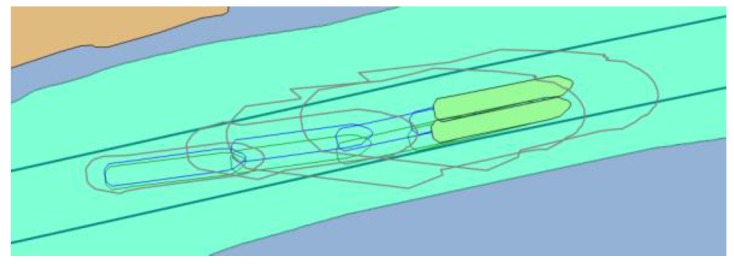
Changes in MVPA size resulting from GNSS signal deterioration.

**Figure 18:**
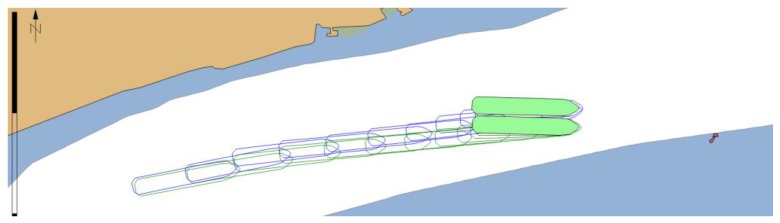

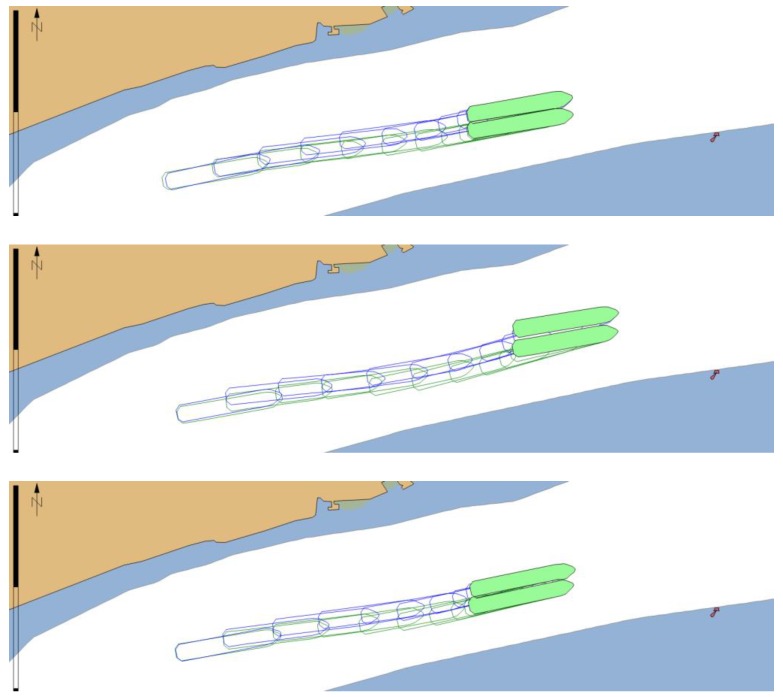
Example of 4 passages of the MASS with simulated GNSS signal deterioration – with integrity data presentation in ECDIS.

When the ECDIS with PNT integrity functionalities was used it presented MVPA and warnings calculated in real time (Figure 17). As a result, the navigator was informed when the position error was too large, and the safe navigation was no longer possible. In this case an emergency stop manoeuvre had to be performed (Figure 18) and the passage could only be continued when the quality of GNSS signal improved.

## 7. Conclusions

The SBAS based model of MASS position sensors data integrity has been elaborated in the paper. The proposed concept of PNT integrity data presentation uses both adaptive safety margins and navigation warning lights. It is based on horizontal protection level model after its modification to elliptical representation and taking into account ship’s dimensions, heading and their accuracy. Vessel technical error limits and virtual approach adaptive channel, whose methodology was based on [30], complement this concept. The first tests confirmed usefulness of the developed methodology. It was proved that in case of the slow deterioration of position accuracy the activation of warning informing about abnormal functioning of position system/sensors would assist navigators in effective actions to avoid grounding.

It is anticipated that such functionalities as GNSS integrity monitoring will soon become compulsory on maritime vessels approved for electronic navigation in coastal waters, narrow passages, harbour approaches, port and limited depth areas and they will be embedded in next generation autonomous surface ships (MASS). However, monitoring of position sensors integrity becomes necessary even nowadays on board classic transport vessels as deliberate spoofing or intentional and incidental jamming is quite probable. Because of these identified vulnerabilities and users’ needs for more reliable position the maritime GNSS integrity standards should be developed taking into account specifics of the elaborated concept. The potential of presented solutions for navigation safety improvement coming from navigator’s better situation awareness (especially for operations performed in restricted visibility) seems to be evident.

The future research should prove benefits of the presented shipborne position sensors’ integrity concept either via further simulation trails in full mission bridge simulator with SBAS functionality [31] or real test-beds.

## Figures and Tables

**Table 1:** HPL for various heading errors estimations.

*s_ψ_* [°]	*HPL* [m]	*HPL_MVPA_* [m]
0.5	11.52	11.55
0.6	11.52	11.56
0.7	11.52	11.58
0.8	11.52	11.61
0.9	11.52	11.63
1.0	11.52	11.67
1.1	11.52	11.71
1.2	11.52	11.76
1.3	11.52	11.82
1.4	11.52	11.89
1.5	11.52	11.98
1.6	11.52	12.08
1.7	11.52	12.21
1.8	11.52	12.37
1.9	11.52	12.55
2.0	11.52	12.77
